# Sex‐Specific Response to Predator Auditory Cues in Asian Corn Borer (*Ostrinia furnacalis*)

**DOI:** 10.1002/ece3.72783

**Published:** 2026-01-05

**Authors:** Li Wang, Qiang Qu, Qiulin Guo, Junhao Guan, Jiayi Zhao, Yue Zhu, Tinglei Jiang, Jiang Feng, Hui Wu

**Affiliations:** ^1^ College of Life Science Jilin Agricultural University Changchun China; ^2^ School of Environment Northeast Normal University Changchun China; ^3^ Jilin Provincial International Cooperation Key Laboratory for Biological Control of Agricultural Pests Jilin Agricultural University Changchun China

**Keywords:** bat ultrasound, ecology of fear, gene expression, *Ostrinia furnacalis*, risk‐induced trait response, sex dimorphism

## Abstract

Predation risk has profound effects on prey from phenotype to gene expression. Prey may respond differently to predation risk on the basis of sex, especially those species with obvious sexual size dimorphism. However, whether such responses are sex‐specific still lacks systematic research. In this study, we continuously exposed a female‐larger species Asian corn borer (ACB, *Ostrinia furnacalis*) to bat foraging ultrasound from the larval stage through adulthood, monitoring phenotypic and gene expression changes in exposed males and females compared to normally reared adults. The results revealed that adults in the ultrasound‐stressed group exhibited significant changes in both phenotypic traits and gene expression profiles, with marked sex‐specific responses to auditory predation cues. Specifically, males demonstrated significant increases in body weight, body length, and ecdysteroid titer, whereas females displayed a marked reduction in fecundity (egg production). Female adults exhibited a predominance of downregulated differentially expressed genes (DEGs), with greater total DEG numbers compared to males. Male adults showed primarily upregulated DEG profiles. Females appear to utilize *LOC114365575* and *LOC114352210* as key regulators in modulating growth and juvenile hormone levels, whereas males may rely on *LOC114349799* and *LOC114351933* to regulate growth and electrophysiological response amplitude under predation risk. Our results suggest that sex‐specific responses may be an important component of inter‐individual differences in prey responses to risk and influence prey population growth and demography.

## Graphical abstract

Females downregulate more DEGs; males upregulate under predation risk. Males boost ecdysteroid; females reduce fecundity under bat ultrasound. Female *O. furnacalis* reacted more strongly to auditory cues of bats than males.

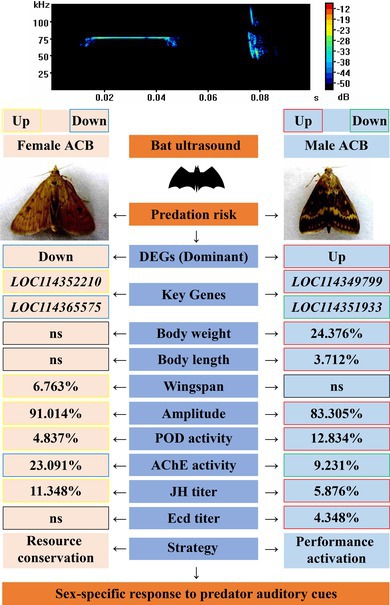

## 1 Introduction

The predator–prey dynamic constitutes a significant element of interspecific interactions (Gramapurohit et al. 2025; Prugh 2023). In addition to direct killing and consumption, the perception of predation risk can also exert indirect effects on prey, known as ‘risk effects’ (Klich et al. 2021; Navarro‐Castilla et al. 2019; Peacor et al. 2020). Although direct predation imposes immediate mortality, studies suggest that the predation risk effect exerts a more profound influence in regulating prey population dynamics and demographic patterns (Gaynor et al. 2019; Gu et al. 2020; Say‐Sallaz et al. 2019; Sheriff et al. 2020; Zanette et al. 2011). When prey perceive predation risk, they typically respond by retreating to refuge habitats and reducing foraging activity (Yager 2012; Zhou et al. 2023). These behavioral changes can, in turn, initiate a cascade of responses spanning from phenotypic to molecular levels (Cinel and Taylor 2019; Liu et al. 2024). These include decreased food intake (Crouchet et al. 2023; Zhou et al. 2023), reduced growth rates (Ball and Baker 1996; Lardner 2000), alterations in body weight (either increase (Davenport et al. 2019) or decrease (Yin et al. 2017)), reduced fecundity (Liu et al. 2024; Tuneu‐Corral et al. 2025; Zhang et al. 2023), fluctuations in stress‐related enzymes (Zha and Lei 2012) and hormones (Monclús et al. 2008; Sapolsky et al. 2000; Zhang et al. 2023), and alterations in gene expression (Berkel and Cacan 2022; Cinel and Taylor 2019; Liu et al. 2024). Examining the multi‐level responses of prey to predation risk, particularly with attention to sex‐specific variation, is critical for elucidating the full scope of risk effects (Cinel and Taylor 2019; Sheriff et al. 2020; Zhang et al. 2023).

Sexual dimorphism in prey morphology (e.g., size, coloration) and behavior (e.g., mating displays) often leads to sex‐biased predation vulnerability (Agan et al. 2024). Divergent reproductive strategies further amplify this dichotomy: males often prioritize mating opportunities through risky behaviors, whereas females invest in offspring quality and self‐maintenance (Winnie and Creel 2007). These life‐history trade‐offs yield distinct risk‐response phenotypes that shape population dynamics (Sheriff et al. 2020) and potential community‐level cascades (Peacor et al. 2020). Yet, the prevailing neglect of intraspecific sexual heterogeneity in the ecology of fear (Nakano et al. 2022; Zanette et al. 2011) obscures a key dimension of predator–prey coevolution.

The bat‐moth predator–prey system has emerged as a paradigmatic model of coevolution, primarily because of the well‐characterized hunting strategies of echolocating bats and their exclusively auditory‐based interactions with moths (Barber et al. 2022). Most predatory bats prey on insects by detecting the accurate position of prey in echolocation (De Conno et al. 2018; Tuneu‐Corral et al. 2023). Ear moth hearing systems, despite their diverse anatomical locations and morphologies, are universally tuned to ultrasonic frequencies (20–60 kHz) for bat detection (Nakano et al. 2014). By sensing bat‐produced ultrasound, moths can escape predators early or even jam their sonar with ultrasonic counter‐signals (Conner and Corcoran 2012). In this evolutionary arms race, bat echolocation calls have paradoxically become a critical acoustic predation cue, triggering anti‐predator responses in moths and creating a predation risk that has intricately shaped their physiology, behavior, and evolutionary trajectory (Göpfert and Hennig 2016). Crambid moths, with their distinctive four‐celled auditory system (A1–A4), grant them acute bat detection sensitivity that facilitates evasion of predation attempts (Nakano et al. 2015). However, most studies on bat‐crambid moth acoustic interactions have focused on behavioral evasion strategies, leaving the potential indirect effects of chronic bat predation risk, such as developmental trade‐offs, reproductive investment shifts, and molecular‐level adaptations, relatively unexplored.

In this study, we investigated sex‐specific phenotypic and molecular responses of the Asian corn borer (*Ostrinia furnacalis*) to predation risk by exposing larvae, pupae, and adults to bat foraging echolocation calls. Given the marked sexual dimorphism in *O. furnacalis*, where females exhibit significantly larger body size and greater reproductive investment than males, we hypothesized that females would demonstrate more pronounced anti‐predator responses across multiple biological levels when exposed to ultrasonic stress. Our comprehensive analysis not only provides novel insights into predator–prey dynamics by revealing sex‐specific adaptive strategies but also highlights the importance of considering sexual dimorphism in ecological and evolutionary studies of anti‐predator responses.

## 2 Materials and Methods

### 2.1 Insects

Eggs of *O. furnacalis* were purchased from Baiyun Industrial Co. Ltd. in Jiyuan, Henan Province, China. Male and female pupae were distinguished on the basis of genital pore position, whereas adult sexes were identified by external characteristics such as body size, weight, coloration, and abdominal morphology. The experimental insects used in this study were from a laboratory colony of *Ostrinia furnacalis* that had been reared for over 20 generations under standardized conditions. This long‐term laboratory rearing promotes genetic stability and homogeneity, which is essential for obtaining reproducible results in both phenotypic and transcriptome sequencing analyses. Larvae were individually reared in 24‐well plates (12.5 cm long × 8.4 cm wide × 2.6 cm high) to prevent cannibalism and ensure accurate tracking. They were fed an artificial diet (1 kg, supplied by Henan Jiyuan Baiyun Industrial Co. Ltd.). The diet composition includes an energy source (wheat germ), a protein source (yeast), gelling agents (konjac flour and carrageenan), preservatives (sorbic acid), as well as vitamin C and lipids (linoleic acid and corn oil). Pupae were individually housed in 25 mL plastic containers (bottom diameter: 3.0 cm, top diameter: 3.8 cm, height: 3.2 cm). After eclosion, adults were kept in plastic cups (10 cm diameter × 10 cm height) and provided with a 10% sucrose solution (500 mL, Shenzhen Xijing Biotechnology Co. Ltd., China) as a nutritional source. All life stages (larvae, pupae, and adults) were maintained in an artificial climate incubator (PRX‐450C, NingboSaifu, Ningbo, China) set at a constant temperature of 27°C, relative humidity of 70%, and a 14:10 h (Light: Dark) photoperiod (with the scotophase from 20:00 to 06:00). Moreover, adults have a tympanic auditory organ containing four auditory cells (A1–A4 cells) and possess a broad auditory frequency range, detecting sounds from 5 to 100 kHz at intensities above 80 dB SPL (Nakano et al. 2015).

### 2.2 Ultrasonic Playback

Bats were captured at the entrance of the cave when they returned from foraging in Shandong Province, China, from July to October 2022. All species were identified on the basis of morphology. The foraging calls were recorded in a makeshift recording laboratory (5 m × 2 m × 2 m) set up in the wild with free‐flying insects as bait. Individual bats (at least 10 individuals of each species) were allowed to fly freely at a time in the acoustics laboratory to record foraging calls, from search‐phase calls to feeding buzzes. The predation process was captured using an infrared camera (FDR‐AX60, Sony Corp, Tokyo, Japan). The foraging ultrasound was recorded using a single‐channel ultrasound recording device (Ultra‐Sound Gate 116, Avisoft Bioacoustics, Berlin, Germany) between 20:00 and 06:00. The sampling frequency was 250 kHz with a 16‐bit resolution. All animal experimental procedures were reviewed and approved by the Animal Ethics Committee of Northeast Normal University, China (Approval No. NENU‐20080416) and the Forestry Bureau of Jilin Province, China (Approval No. JLFB‐[2006]‐178).

The playback stimuli in this study were synthesized from the echolocation calls of the constant frequency of bat *Rhinolophus ferrumequinum* and the frequency modulation of bat *Myotis fimbriatus* . The acoustic stimuli consisted of a combination of long‐duration constant‐frequency pulses and short‐duration frequency‐modulated pulses. This specific combination was selected because our preliminary screening of various ultrasonic signals indicated that it triggered the most robust and comprehensive anti‐predator responses in *O. furnacalis*, including significant acetylcholinesterase activity suppression in male pupae and female adults (see Figure S1). This is consistent with the ecological rationale that a complex signal, mimicking a diverse bat assemblage, represents a heightened level of threat (Nakano et al. 2015). The echolocation calls of these two species were stitched together using the Avisoft‐SASLab Pro 5.2 (Avisoft Bioacoustics, Berlin, Germany) software to construct a playback file of 1 min. Each playback file comprised 10 different call sequences with silent intervals of approximately 5 s between sequences to simulate the natural intervals of echolocation calls. The experimental group (EG) was exposed to ultrasound throughout the life cycle every night (20:00–06:00) from the first‐instar larval stage through the adult stage using an ultrasound playback device (UltraSoundGate Player 116, Avisoft Bioacoustics, Berlin, Germany), whereas the control group (CG) was cultured under the same conditions but without ultrasonic playback. The playback group maintained a sound intensity of 100 dB peSPL, monitored with a sound level meter (DSM D1, DELIXI ELECTRIC, China) and regulated via an ultrasonic dynamic speaker (Avisoft Bioacoustics) at a 1 cm distance (Liu et al. 2024). The prolonged exposure protocol was employed to model ecological scenarios of sustained predation risk, such as in areas of high bat density. This approach was essential for eliciting measurable, cumulative effects on phenotypic traits and gene expression, which are the central focus of this mechanistic study.

### 2.3 Phenotypic Monitoring During Playback Experiment

When the larvae developed into the adult stage, their morphological traits (body weight, length, wingspan), neurophysiological parameters (action potential amplitude of adults' auditory neurons recorded extracellularly), hormone levels (juvenile hormone and ecdysone), and enzyme activities (acetylcholinesterase and peroxidase) were quantified in both sexes. Each group included 100 males and 100 females for phenotypic evaluation, with fecundity records for females.

The body weight was measured using a digital electronic balance (AL104, 110 g/0.1 mg, Mettler‐Toledo Instruments (Shanghai) Co. Ltd., China). Body size was determined using electron digital calipers (DEGUQMNT, Meinet Group, Germany) for body length and wingspan (Salcedo et al. 2023). The electrophysiological signals of auditory neurons of every adult were recorded digitally from two channels to a computer (QitianM410‐D214, Lenovo Group, China) for at least 10 min using the Spike Recorder software and Neuron SpikerBox (Neuron SpikerBox Pro, Backyard Brains, America) (Limnuson et al. 2015). The Spike Recorder software was used to process the data to evaluate amplitude, inter‐spike interval time, nerve conduction velocity, and response frequency on the recorded electrophysiological signal files after the recording. The action potential amplitude of adults' auditory neurons recorded extracellularly was selected as the primary indicator to quantify the electrophysiological signals. The action potential amplitude of adults' auditory neurons recorded extracellularly represents the maximum value of electrophysiological signals and is also called the peak value (Yeldesbay et al. 2024).

The double‐antibody sandwich enzyme‐linked immunosorbent assay (Sangon Biotech (Shanghai) Co. Ltd., China) was performed to evaluate juvenile hormone (JH) and ecdysone (Ecd) titers and acetylcholinesterase (AChE) and peroxidase (POD) activities in the adults (Guo et al. 2017).

For each phenotypic trait, the Sexual Dimorphism Index (SDI) was calculated separately. The SDI formula is: SDI = (F−M)/M, where F represents the measured value of the specific trait in females, and M denotes the corresponding value in males (Santoni et al. 2023).

Newly emerged (1‐day‐old) female and male moths were paired in plastic cups lined with plastic bags for egg collection. Female fecundity (total eggs laid per female until death) was quantified under a stereoscopic microscope (Motic, China). Sample size was more than 10 pairs per treatment (Iqbal et al. 2021).

### 2.4 Transcriptome Sequencing

Newly emerged (1‐day‐old) ACB adults were used for transcriptome sequencing, with three replicates for each sex per treatment. The experimental group's females and males were designated as EF and EM, respectively, whereas the control group counterparts were labeled CF and CM. All samples of the whole body of adults were quickly quenched in liquid nitrogen and then stored in the −80°C refrigerator. Adult mRNA of the Asian corn borer was extracted using the Trizol reagent (Invitrogen, USA). mRNA was tested using Nanodrop (OD 260/280). Transcriptome sequencing was performed on the Illumina Novaseq 6000 sequencing platform, with the sequencing mode being PE150. Then the transcriptome de novo assembly and the differentially expressed genes were analyzed (Luo et al. 2025).

### 2.5 Analysis of the Differentially Expressed Genes

Differential gene expression analysis was performed using the R package DESeq2 (v1.12.4) with significance thresholds set at *q* < 0.05 and |FoldChange| > 6 (Rosati et al. 2024). Functional enrichment analysis of differentially expressed genes (DEGs) was conducted using clusterProfiler (v3.0.5) for GO (Gene Ontology), KEGG, and KOG classifications, with statistical significance defined as *q* < 0.05 (Fedorov et al. 2025). A higher rich factor and a lower *q*‐value indicate a stronger enrichment. Only the top 30 most significantly enriched pathways are shown (The Gene Ontology Consortium et al. 2023).

We performed weighted gene co‐expression network analysis (WGCNA) using the R package WGCNA (version 1.51) on differentially expressed genes. Gene module‐trait relationships were evaluated by Pearson correlation analysis, with absolute correlation coefficients |*r*| > 0.8 and *p* < 0.05 considered statistically significant (Langfelder and Horvath 2008).

### 2.6 Construction of the PPI Network and Screening of Key Genes

Gene significance (GS) and module membership (MM) were calculated for each gene within the respective modules. In modules significantly associated with phenotypes, genes with |GS| > 0.9 and |MM| > 0.9 were identified as hub genes. The selected hub genes were then imported into the STRING database (http://string‐db.org/) to construct a protein–protein interaction (PPI) network. Using the cytoNCA plugin in Cytoscape 3.9.1, the Degree values of PPI network nodes were calculated, and the top 20 nodes (or all if fewer than 20) were visualized. On the basis of Degree values and gene functions, the final key genes for each module were screened (Li et al. 2025).

### 2.7 Statistical Analysis

The normal distribution of the data was evaluated using the Kolmogorov–Smirnov test. The homogeneity of variance of normally distributed data was examined. Normally distributed data with homogeneous variance were compared using a *T*‐test for the independent samples. Non‐normally distributed data without homogeneous variance were compared using the Kruskal–Wallis. All statistical analyses were performed using the R (version 4.5.0) software (Kadiyala and Kumar 2017).

## 3 Results

### 3.1 Phenotypic Indicators of Asian Corn Borer in Response to Ultrasonic Stress

**FIGURE 1:**
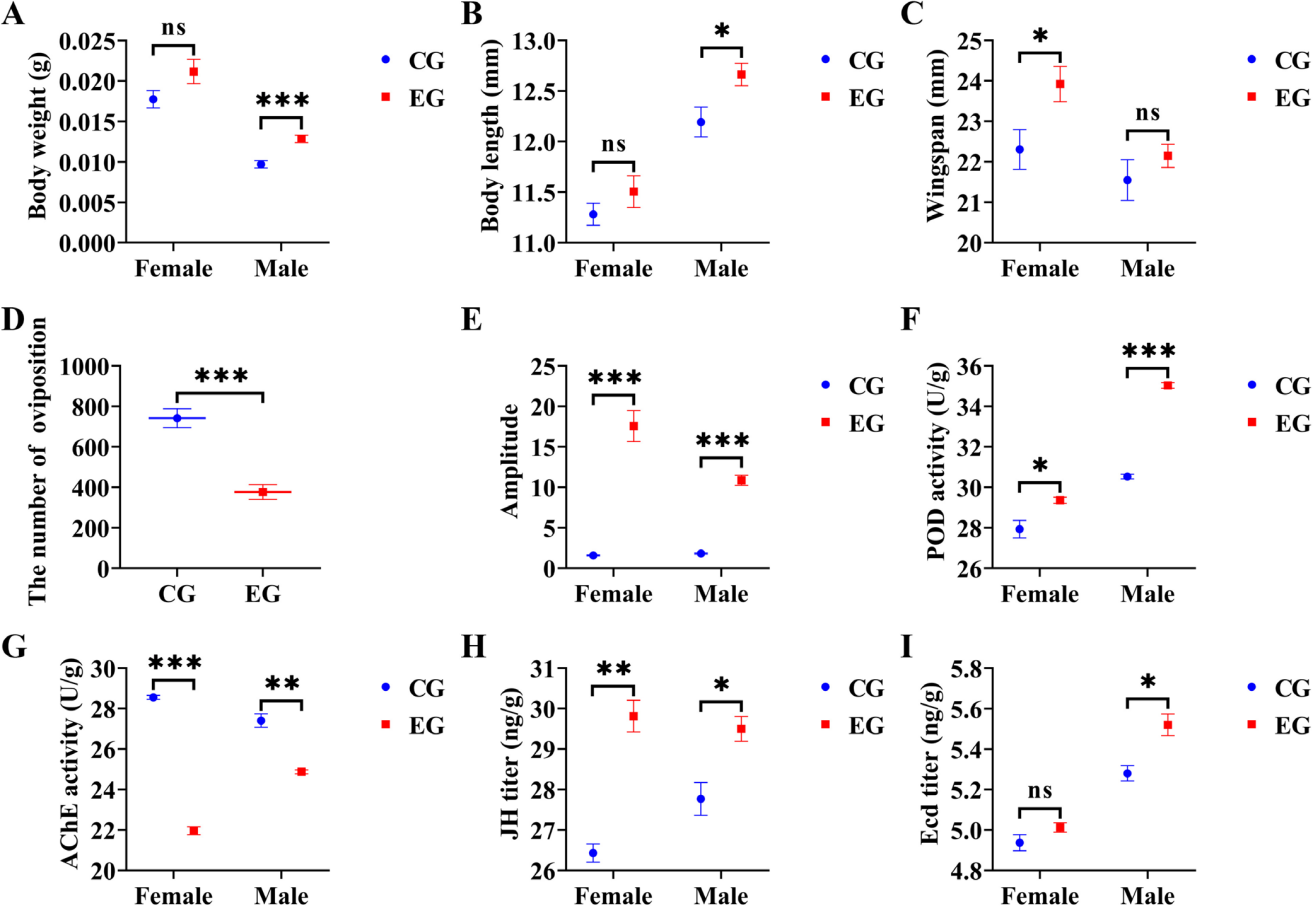
The differences in male and female phenotypes between the experimental group and the control group. Results are expressed as mean ± standard error, ns indicates not significant (*p* > 0.05), *p ≤ 0.05, **p ≤ 0.01, ***p ≤ 0.001.

**FIGURE 2:**
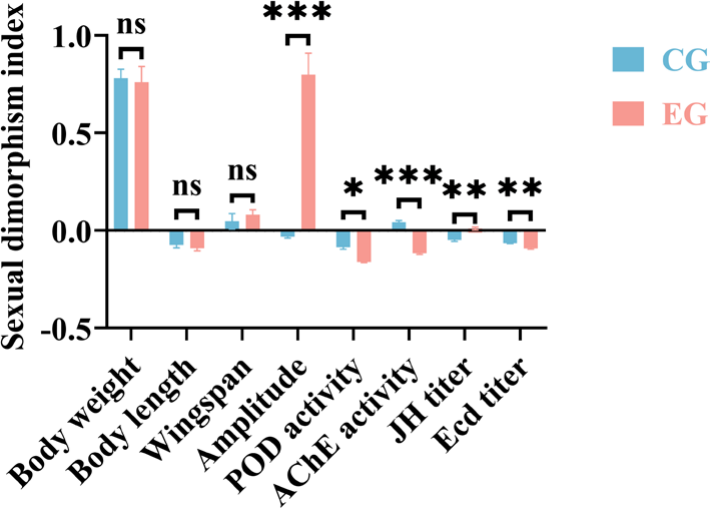
Differences in sexual dimorphism index between the experimental group and the control group. Results are expressed as mean ± standard error, ns indicates not significant (*p* > 0.05), *p ≤ 0.05, **p ≤ 0.01, ***p ≤ 0.001.

When chronically exposed to bat foraging ultrasound from the first‐instar larval stage through adulthood, male adults exhibited a 24.376% increase in body weight (*t* = 3.878, *p* < 0.001; Figure 1A), a 3.712% increase in body length (*t* = 2.565, *p* = 0.012; Figure 1B). Female adults showed a 6.763% increase in wingspan (*t* = 2.411, *p* = 0.018; Figure 1C) and a 49.171% reduction in fecundity (*t* = 6.114, *p* < 0.001; Figure 1D). The amplitude of electrical signals increased significantly by 91.014% in females and 83.305% in males (*Z* = 6.653, *p* < 0.001; *Z* = 7.698, *p* < 0.001; Figure 1E). Additionally, the sexual dimorphism index of this parameter rose significantly by 1.040‐fold (*Z* = 6.653, *p* < 0.001; Figure 2). The increase in peroxidase activity was more pronounced in males compared to females (Figure 1F). Consequently, the sexual dimorphism index of this parameter decreased significantly by 47.407% (*t* = 7.000, *p* = 0.016; Figure 2). The activity of AChE in female adults exhibited a more pronounced decrease compared to males (Figure 1G). JH titers in adult males and females increased significantly by 11.348% (*t* = 7.494, *p* = 0.002) and 5.876% (*t* = 3.404, *p* = 0.027), respectively (Figure 1H), accompanied by a 5.515‐fold elevation in the sexual dimorphism index (*t* = 5.385, *p* = 0.006; Figure 2). In males, the ecdysone titer showed a significant 4.348% increase (*t* = 3.689, *p* = 0.021; Figure 1I), whereas no statistically significant difference was observed in females (*t* = 1.677, *p* = 0.169; Figure 1I).

### 3.2 Differentially Expressed Genes of Asian Corn Borer in Response to Ultrasonic Stress

**FIGURE 3:**
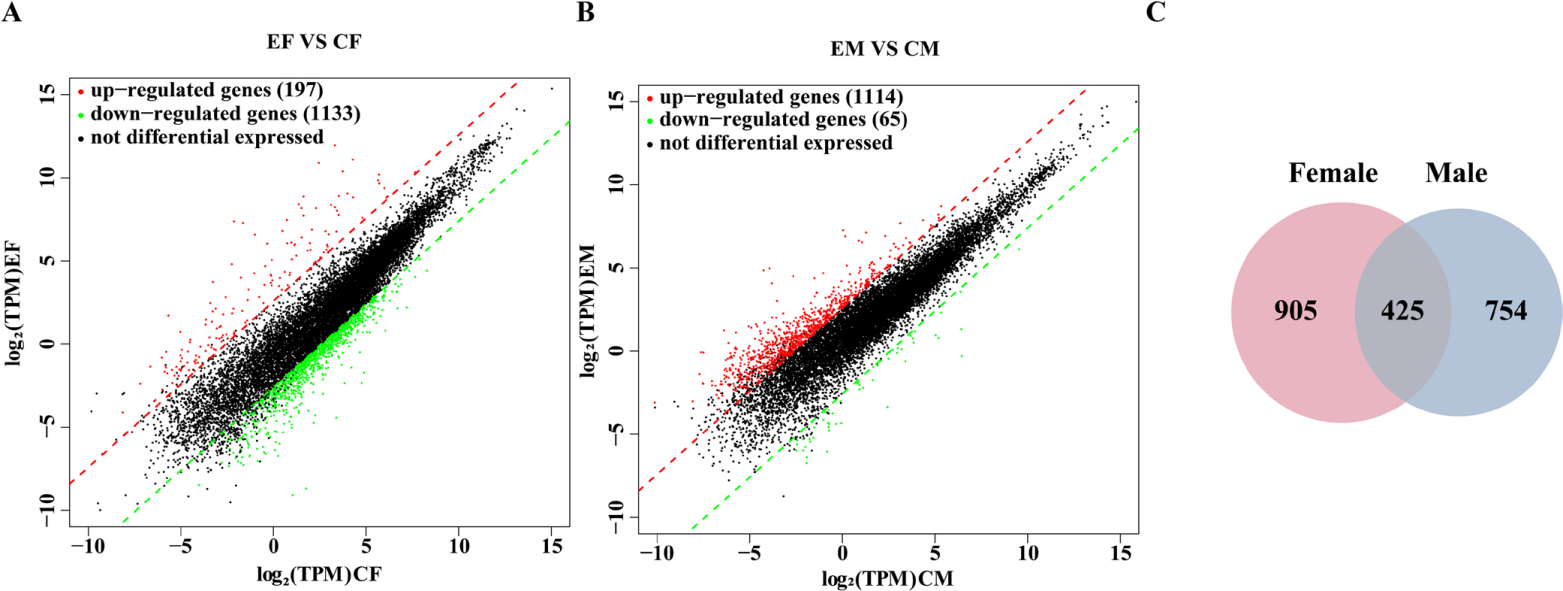
Scatter plots of differentially expressed genes in (A) females and (B) males between the experimental group and the control group, and (C) a Venn diagram. Up‐ and downregulated genes are shown in red and green, respectively.

Under ultrasound stress conditions, females exhibited a total of 1330 differentially expressed genes (DEGs), of which 197 were upregulated (accounting for 15%), and 1133 were downregulated (accounting for 85%). Male adults showed 1179 DEGs, with 1114 upregulated (94%) and 65 downregulated (6%). Females displayed a higher number of DEGs in response to predation risk than males (Figure 3A,B). Moreover, the DEGs in females were predominantly downregulated, whereas those in males were mainly upregulated. Among the DEGs, 425 were shared between females and males, whereas 905 were female‐specific and 754 were male‐specific. Most of the DEGs detected in females under predation risk were not observed in males (Figure 3C).

### 3.3 Enrichment Analysis Results of DEGs

**FIGURE 4:**
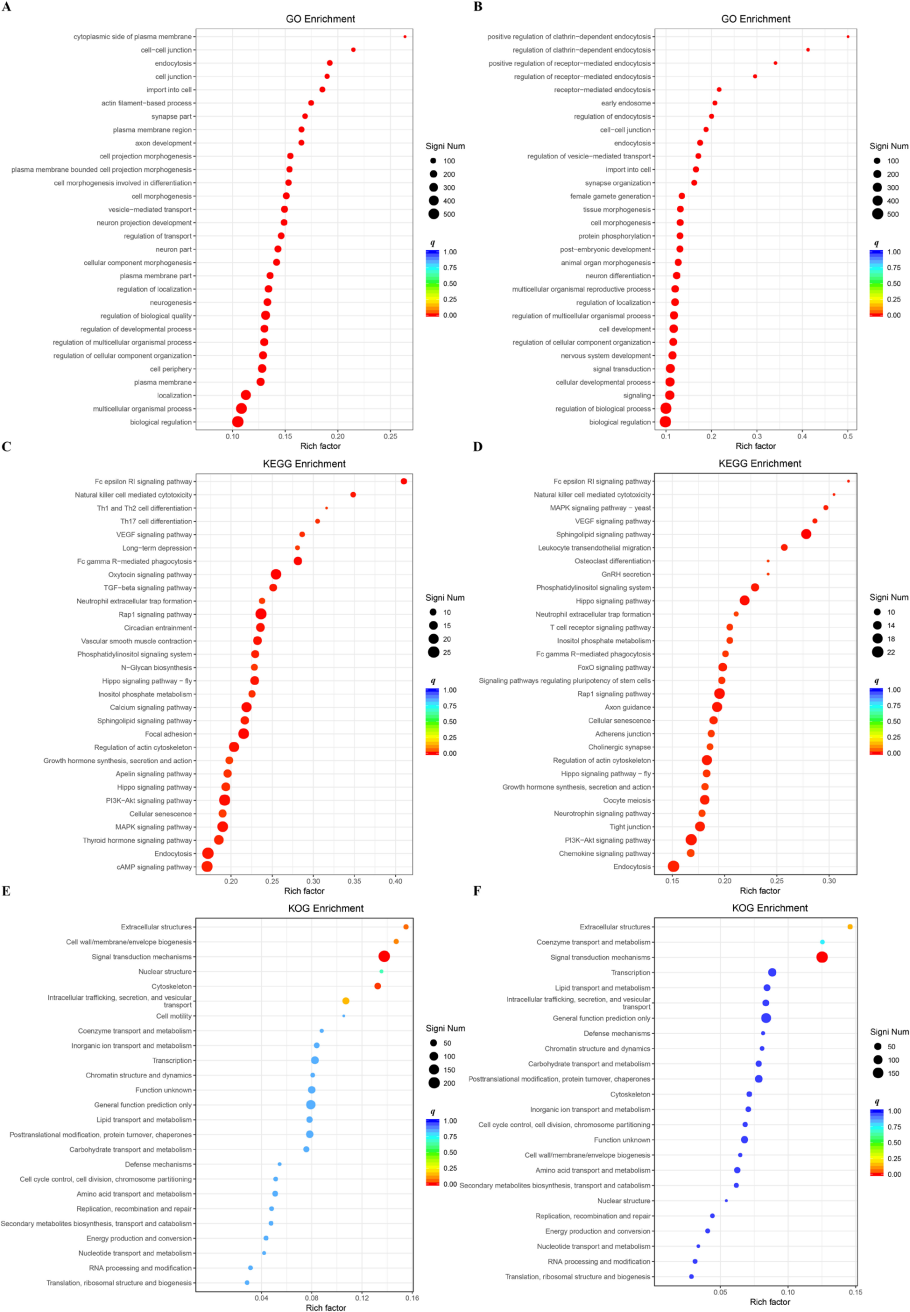
Functional enrichment analysis of differentially expressed genes for females (A, C, E) and males (B, D, F), respectively. The rich factor, defined as the ratio of differentially expressed genes to total genes annotated in a pathway, and the adjusted *q*‐value were used to evaluate enrichment significance.

The GO enrichment analysis results demonstrated that in males, a substantial number of DEGs were enriched in reproduction‐related biological processes, including the multicellular organismal reproductive process. In both female and male adults, numerous DEGs were significantly enriched in growth and development‐related processes, specifically the regulation of developmental processes in females and cell development and cellular developmental processes in males (Figure 4A,B).

KEGG pathway analysis revealed that females exhibited significant enrichment of DEGs in reproduction‐associated pathways, notably the oxytocin signaling pathway. Additionally, both sexes showed considerable enrichment of DEGs in growth‐related pathways, with prominent representation in growth hormone synthesis, secretion, and action (Figure 4C,D).

KOG functional analysis identified signal transduction mechanisms (category T, *q* < 0.001) as the only significantly enriched functional category shared between female and male adults. Moreover, multiple DEGs in both sexes were functionally linked to acetylcholinesterase‐related activities, including the classification KOG4389—acetylcholinesterase/Butyrylcholinesterase [T] (Figure 4E,F).

### 3.4 Linking Co‐Expression Modules to Phenotypic Responses Under Predation Risk

**FIGURE 5:**
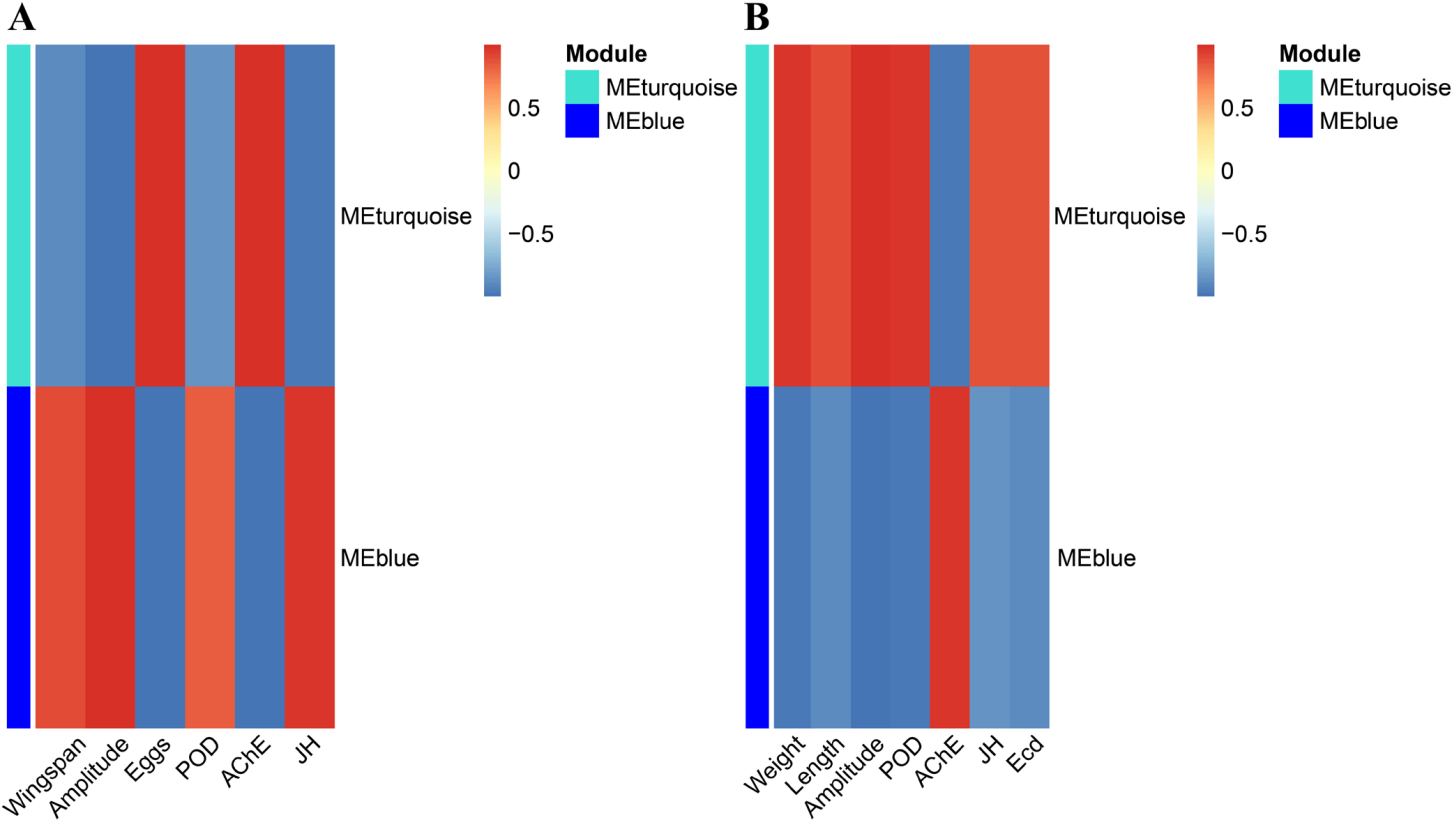
Heatmap of correlations between key modules and phenotypes. (A) Females, (B) Males. Red/blue colors indicate positive/negative correlations. Measured parameters: Wingspan, body weight (weight), body length (length), fecundity (eggs), action potential amplitude (amplitude), juvenile hormone titer (JH), ecdysone titer (Ecd), acetylcholinesterase activity (AChE), and peroxidase activity (POD).

In both females and males, WGCNA identified two co‐expression network modules: MEturquoise and MEblue. In females, genes within the MEturquoise module were uniformly downregulated. This module exhibited significant positive correlations with AChE (*r* = 0.996, *p* < 0.001) and female fecundity (*r* = 0.983, *p* < 0.001), while showing significant negative correlations with electrophysiological signals amplitude (*r* = −0.986, *p* < 0.001), JH (*r* = −0.965, *p* = 0.002), POD (*r* = −0.852, *p* = 0.031), and wingspan (*r* = −0.898, *p* = 0.015). Conversely, genes in the MEblue module were upregulated and displayed strong positive correlations with electrophysiological signals amplitude (*r* = 0.988, *p* < 0.001), JH (*r* = 0.960, *p* = 0.002), POD (*r* = 0.828, *p* = 0.042), and wingspan (*r* = 0.899, *p* = 0.015), but negative correlations with AChE (*r* = −0.989, *p* < 0.001) and fecundity (*r* = −0.985, *p* < 0.001) (Figure 5A).

In males, the MEturquoise module genes were upregulated and positively correlated with amplitude (*r* = 0.989, *p* < 0.001), Ecd levels (*r* = 0.865, *p* = 0.026), JH (*r* = 0.869, *p* = 0.025), body length (*r* = 0.896, *p* = 0.016), POD (*r* = 0.963, *p* = 0.002), and weight (*r* = 0.962, *p* = 0.002), whereas negatively correlated with AChE (*r* = −0.961, *p* = 0.002). In contrast, MEblue module genes were downregulated, showing a positive correlation with AChE (*r* = 0.962, *p* = 0.002) but negative correlations with amplitude (*r* = −0.995, *p* < 0.001), Ecd (*r* = −0.883, *p* = 0.020), JH (*r* = −0.854, *p* = 0.030), body length (*r* = −0.895, *p* = 0.016), POD (*r* = −0.970, *p* = 0.001), and weight (*r* = −0.965, *p* = 0.002) (Figure 5B).

These results highlight the MEturquoise and MEblue modules as key regulatory hubs in both female and male adults, exhibiting sex‐specific expression patterns and opposing correlations with physiological traits critical to growth, reproduction, and stress response.

### 3.5 Construction of PPI Networks and Identification of Key Genes

**FIGURE 6:**
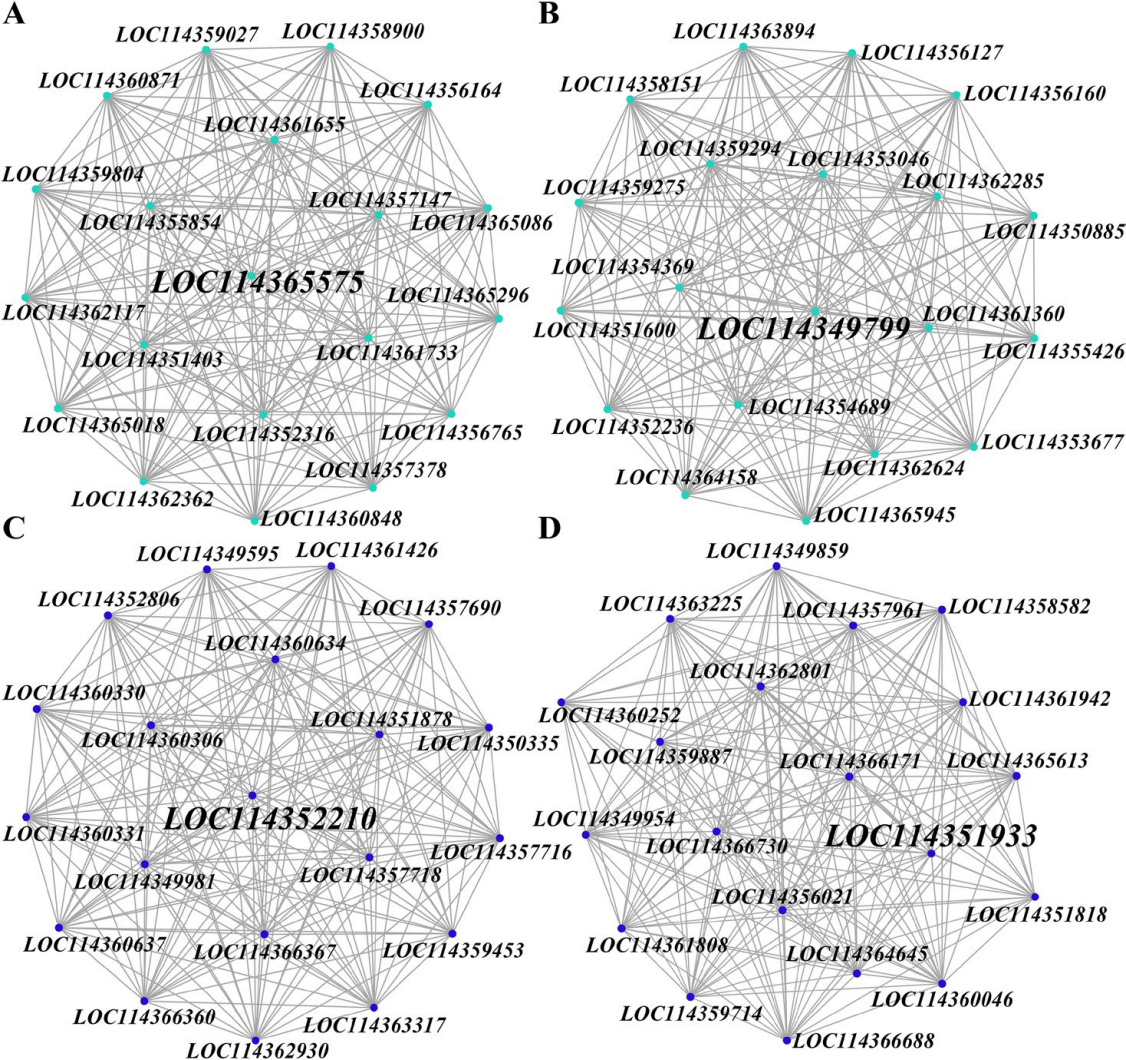
Protein‐protein interaction (PPI) networks of key module genes. (A) Females (MEturquoise), (B) Males (MEturquoise), (C) Females (MEblue), (D) Males (MEblue). Nodes represent genes, and edges indicate significant co‐expression relationships (|Pearson correlation| > 0.8, *p* < 0.05). Hub genes are highlighted in bold.

PPI network construction identified 20 hub genes from each key module. In the female ME turquoise module, the key gene associated with ultrasound‐induced phenotypic changes was *LOC114365575* (annexin B9‐like isoform X1) (Figure 6A). In the corresponding male module, the gene linked to ultrasound‐induced phenotypic alterations was *LOC114349799* (septin‐7 isoform X1) (Figure 6B). In the female MEblue module, the gene associated with ultrasound‐induced phenotypic changes was *LOC114352210* (juvenile hormone esterase‐like) (Figure 6C), whereas in the male MEblue module, it was *LOC114351933* (annotated as K01312‐ko04080: Neuroactive ligand‐receptor interaction) (Figure 6D).

Therefore, when facing acoustic predation risk from bats, female Asian corn borer likely utilize *LOC114365575* and *LOC114352210* as key players, whereas males may rely on *LOC114349799* and *LOC114351933* to mediate critical responses.

## 4 Discussion

### 4.1 Predation Risk Elicited Sex‐Specific Responses in *O. furnacalis*

The central theme of this study is that bat foraging ultrasounds, serving as a predation risk cue, act as a differential trigger for sex‐specific responses in the Asian corn borer. The results support our hypothesis regarding sex‐specific responses to predation risk. Females primarily exhibit phenotypic and gene expression changes related to reproduction. In contrast, males show responses predominantly in gene expression linked to neural signaling and cellular growth and development.

We observed a distinct sex‐specific pattern in the transcriptional response to predation risk. In males, the majority of differentially expressed genes (DEGs) were upregulated. A key example is the gene encoding septin‐7 isoform X1 (*LOC114349799*), which is implicated in cell development and cellular developmental processes. This upregulation suggested a potential mechanistic link to the observed changes in body weight and length, a finding consistent with the role of septins in growth regulation (Vagin and Beenhouwer 2016). Conversely, the female transcriptional profile was predominantly characterized by downregulation. A representative case is the annexin B9‐like isoform X1 (*LOC114365575*), a gene whose suppressed expression may influence the regulation of developmental processes. This modulation is potentially linked to altered cell growth, proliferation, and differentiation, ultimately affecting wing development, as supported by previous studies on annexin functions (Wu et al. 2014).

Upon exposure to ultrasonic stimuli, female oviposition was significantly suppressed (Figure 1D). This suppression may be attributed to predator‐induced stress, which disrupts resource allocation, compromises oxidative stress recovery, and impairs overall physiological homeostasis, collectively contributing to reproductive deficits (Corcoran et al. 2013; Magnhagen 1991; Mukherjee et al. 2014; Zanette et al. 2011). In support of this, KEGG enrichment analysis revealed that female DEGs were significantly enriched in reproduction‐related pathways, most notably the oxytocin signaling pathway. The altered expression of genes within this pathway likely further disrupts reproductive physiology (Cinel and Taylor 2019; Liu et al. 2024).

Meanwhile, KOG4389—acetylcholinesterase/butyrylcholinesterase [T] was significantly enriched in both male (*LOC114351933*) and female adults. This enrichment may reduce acetylcholinesterase activity, thereby impairing the normal hydrolysis of the neurotransmitter acetylcholine. As a result, the persistent excitation of nerve cells could lead to an increase in the amplitude of action potentials (Liang et al. 2025). Concurrently, upregulation of reactive oxygen species (ROS)‐related genes indicated elevated oxidative pressures. In response, peroxidase‐associated genes were also upregulated, likely enhancing peroxidase activity to mitigate oxidative damage and establish an effective antioxidant defense (Zha and Lei 2012).

At the hormonal level, female moths exhibited upregulation of a key juvenile hormone (JH)‐related gene (*LOC114352210*), which is involved in the growth hormone synthesis, secretion, and action pathway. This transcriptional change suggested a potential increase in JH titers, a response that may be linked to resource allocation under predation risk (Cheng and Zhou 2025). In contrast, males showed enhanced expression of hormone‐related genes, which were also associated with the growth hormone synthesis, secretion, and action pathway. This coordinated upregulation likely elevates both JH and ecdysteroid levels, reflecting a distinct endocrine strategy in response to predation risk (Ables et al. 2015; Chaitanya et al. 2013; Valzania et al. 2016).

### 4.2 Ecological and Evolutionary Implications of Sex‐Specific Responses

Predation risk can drive phenotypic divergence between sexes, a phenomenon often mediated by sex‐specific life‐history strategies (Donelan and Trussell 2020). Our study reveals transcriptional differences underpinning such sex‐specific strategies, which align with findings in *Marmota flaviventer*, supporting the generality of sex‐dependent responses to predation risk (Berkel and Cacan 2022). These divergent responses likely arise from distinct evolutionary pressures and life‐history trade‐offs: as the primary reproductive investors, females may prioritize long‐term survival and reproductive success, thus adopting more conservative strategies to maintain homeostasis and preserve future reproductive potential under predation risk (Liu et al. 2024; Magnhagen 1991). In contrast, males may employ more physiologically activated strategies that emphasize immediate escape capacity and mating opportunities (Lima and Dill 1990; Zhang et al. 2023). Such strategic divergence is critical for understanding population adaptability, resilience, and dynamics in predation‐risk environments (Berkel and Cacan 2022). Therefore, our study reveals sex‐specific responses at multiple levels, from gene expression to phenotype, which provide new insights into predation risk effects and the ecology of fear under uniform predation risk (Berkel and Cacan 2022; Peacor et al. 2020).

### 4.3 Limitations of Study and Future Research Directions

Under controlled laboratory conditions, this study maintained a constant duration and intensity of predation risk, which may differ from the unpredictable nature of risk encounters in natural settings. Therefore, our findings primarily elucidate prey adaptation patterns under sustained risk exposure. Future studies should validate these results in more complex natural environments and investigate how fluctuating or intermittent risk dynamically shapes prey adaptive strategies.

Given that our experimental design involved exposing the Asian corn borer to bat ultrasound from the larval through pupal to adult stages, the auditory capacity of the pre‐adult stages warrants consideration. Lepidopteran larvae, however, lack tympanic auditory organs. Evidence suggests that their sound perception may be mediated by filiform hairs or other mechanosensory structures that detect acoustic vibrations (Agah‐Manesh et al. 2021; Taylor and Yack 2019). For instance, *Danaus plexippus* larvae use proprioceptors on their prothorax (Taylor and Yack 2019), and larvae of other species like *Barathra brassicae* possess thoracic setae responsive to sound stimulation (Markl and Tautz 1975; Tautz 1977). The high sound pressure levels of bat echolocation calls may directly stimulate mechanosensory structures (e.g., verruca) in Asian corn borer larvae, potentially eliciting pronounced anti‐predator responses consistent with perceived predation risk (Lee et al. 2021; Shams Salehi et al. 2016). The perception of ultrasound at the pupal stage is likely mediated by the setae located on the pupal terminal segments of the Asian corn borer (Field and Matheson 1998; Hill 2009; Zhou et al. 2023). Future studies employing neuroanatomical and electrophysiological techniques are urgently needed to map the vibration‐sensitive regions and intrinsic pathways that transduce acoustic cues into physiological responses in both larvae and pupae.

Beyond the documented within‐generation effects, the potential for these ultrasound‐induced responses to persist intergenerationally warrants investigation. Future work should therefore examine the offspring of exposed adults for inherited changes in stress resilience, development, and reproduction. Quantifying key fitness indicators across the subsequent generation's life cycle is essential to model the relationship between acoustic exposure, offspring performance, and population trajectories. Such a model is critical for forecasting the long‐term efficacy of ultrasonic pest control (Hermann et al. 2021).

A key laboratory observation was the dramatic decline (~49%) in fecundity in females exposed to bat echolocation calls (Figure 1D), revealing an intrinsic vulnerability in pest population growth. This offers a theoretical foundation for developing acoustic‐based, green pest control strategies that target reproductive behavior. Our findings reveal a novel pest management strategy: using simulated bat calls to disrupt reproduction, which capitalizes on the female‐specific physiological responses identified in this study. Although practical field application will require addressing challenges related to technical feasibility, environmental adaptation, and cost‐effectiveness, this approach aligns closely with sustainable agricultural goals by aiming to suppress pest populations at the source while reducing reliance on chemical insecticides (Tuneu‐Corral et al. 2025).

## 5 Conclusion

Our study demonstrates that chronic exposure to bat predation ultrasound induces sex‐specific phenotypic and transcriptomic responses in the female‐larger Asian corn borer (*Ostrinia furnacalis*), revealing critical divergences in how males and females allocate resources under predation risk. Although males prioritized somatic growth (increased body weight, length, and ecdysteroid titers), females exhibited significant reproductive trade‐offs (reduced fecundity). Transcriptomic analyses further underscored this dichotomy: females showed predominant downregulation of genes linked to juvenile hormone signaling and growth regulation, whereas males upregulated genes associated with electrophysiological response and development. Sex‐specific candidate regulators suggest distinct molecular pathways mediate these adaptive strategies. Our study provided fundamental insights into how predation pressure drives the divergence of life‐history strategies in sexually dimorphic species.

## Author Contributions

**Li Wang:** data curation (equal), formal analysis (equal), investigation (equal), methodology (equal), software (equal), visualization (equal), writing – original draft (equal), writing – review and editing (equal). **Qiang Qu:** data curation (equal), investigation (equal), visualization (equal). **Qiulin Guo:** data curation (equal), investigation (equal). **Junhao Guan:** data curation (equal), investigation (equal). **Jiayi Zhao:** data curation (equal), investigation (equal). **Yue Zhu:** investigation (equal). **Tinglei Jiang:** conceptualization (equal), methodology (equal). **Jiang Feng:** conceptualization (equal), funding acquisition (equal), methodology (equal). **Hui Wu:** conceptualization (equal), funding acquisition (equal), methodology (equal), project administration (equal), supervision (equal), validation (equal), writing – review and editing (equal).

## Funding

This work was supported by the Jilin Provincial Natural Science Foundation (Grant No. 20250102310JC), Changbai Yingcai Program‐ Young Top‐notch Researchers Project (Grant No. 202441131), National Natural Science Foundation of China (32171489) and Jilin Provincial Science and Technology Development Program No. 20230502003GH.

## Conflicts of Interest

The authors declare no conflicts of interest.

## Supporting information


**Data S1:** Supporting Information.

## Data Availability

All relevant data are contained within this research article and in the Supporting Information.
